# Intravoxel incoherent motion MRI as a biomarker of sorafenib treatment for advanced hepatocellular carcinoma: a pilot study

**DOI:** 10.1186/s40644-016-0059-3

**Published:** 2016-01-29

**Authors:** Natsuhiko Shirota, Kazuhiro Saito, Katsutoshi Sugimoto, Kenichi Takara, Fuminori Moriyasu, Koichi Tokuuye

**Affiliations:** Department of Radiology, Tokyo Medical University, 6-7-1 Nishishinjuku, Shinjuku-ku, Tokyo 160-0023 Japan; Department of Gastroenterology and Hepatology, Tokyo Medical University, Shinjuku-ku, Tokyo Japan

**Keywords:** Sorafenib, Hepatocellular carcinoma, Intravoxel incoherent motion, Biomarker, Diffusion-weighted imaging

## Abstract

**Background:**

To evaluate the association between the therapeutic outcomes of sorafenib for advanced hepatocellular carcinoma (HCC) and the parameters of intravoxel incoherent motion (IVIM).

**Methods:**

Nine patients were evaluated prospectively. All patients were Child-Pugh score A. The mean dimension of the lesion was 32 mm (range: 15–74 mm). MR images were obtained using a 1.5-Tesla superconductive MRI system. Diffusion-weighted imaging was performed under breath-holding using *b*-values of 0, 50, 100, 150, 200, 400, and 800 s/mm^2^. The following IVIM parameters were calculated: apparent diffusion coefficient, true diffusion coefficient (DC), pseudo-diffusion coefficient, and perfusion fraction. MRI was performed before treatment and at 1, 2, and 4 weeks after beginning treatment. Tumor response at 4 weeks was assessed by CT or MRI using modified RECIST. IVIM parameters of the treatment responders and non-responders were compared.

**Results:**

The DC of responders at baseline was significantly higher than that of the non-responders. The sensitivity and specificity, when a DC of 0.8 (10^−3^ mm^2^/s) or higher was considered to be a responder, were 100 % and 67 %, respectively. No significant differences were found in the other parameters between the responders and the non-responders. All IVIM parameters of the responders and non-responders did not change significantly after treatment.

**Conclusion:**

The DC before treatment may be a useful parameter for predicting the therapeutic outcome of sorafenib for advanced HCC.

## Background

The multikinase inhibitor sorafenib was reported to prolong the median survival and time to progression of patients with advanced hepatocellular carcinoma (HCC) [1]. Sorafenib inhibits tumor-cell proliferation and tumor angiogenesis [2]. This drug prolongs the stable state of HCC by reducing blood flow to the tumor and by increasing tumor-cell apoptosis, rather than by decreasing tumor size [1]. However, it was reported that the therapeutic effect of sorafenib could not be accurately evaluated using the Response Evaluation Criteria in Solid Tumors (RECIST) [3], which is conventionally used. Therefore, it has been proposed that the modified RECIST, including the effect on blood flow is more useful for the evaluation of the therapeutic outcomes of cancers [4, 5].

On the other hand, other studies have concluded that diffusion-weighted imaging (DWI) was useful for the evaluation of the therapeutic outcomes of advanced HCC, and did not require data on arterial blood flow in the lesion [6–8]. Furthermore, it has been proposed that DWI is effective for evaluating the therapeutic outcomes of chemotherapy or radiation therapy on other types of tumors [9–11]. DW images are obtained by visualizing the motion of water molecules randomly using MRI. Because DWI is sensitive to changes of intracellular substances and cell membranes, it is often used for therapeutic monitoring [10]. In previous reports, the bleeding or necrosis of tumors increased diffusion values in the responder group [6].

Lewin et al. reported the usefulness of intravoxel incoherent motion (IVIM) for the evaluation of the therapeutic outcome of sorafenib [7]. It is possible to obtain the true diffusion coefficient reflecting cell density and the perfusion fraction reflecting the microcirculation of tumors using IVIM. Therefore, IVIM may reflect tumor necrosis and neovascular inhibition resulting from the therapeutic effect of sorafenib. Furthermore, IVIM may be able to predict therapeutic outcomes before treatment. Because sorafenib treatment frequently causes side effects and is expensive, it is often difficult for patients to maintain medication compliance. Therefore, it would be highly beneficial if therapeutic efficacy could be determined at an early stage.

Here we reported a pilot study on the efficacy of IVIM for the evaluation of the therapeutic effects and early treatment effects of sorafenib for advanced HCC.

## Methods

The study was approved by the ethics committee of our institution, and written informed consent was obtained from all the patients who participated in this study.

### Subjects

Thirty-seven patients with HCC were examined from July 2009 to January 2012. The study inclusion criteria were patients receiving sorafenib therapy, Barcelona Clinic Liver Cancer stage of B or C, and no contraindications to MRI. This prospective study was part of an assessment of the efficacy of radiological analysis to predict therapeutic outcomes and prognostic expectations. Radiological assessment included contrast-enhanced ultrasound and MRI. Some patients refused all 3 MRI examinations or carelessly forgot to undergo an examination, and 10 patients remained in the study. Of the 10 patients who underwent liver MRI, 1 was excluded because of poor image quality due to artifacts. The final study population included 9 patients with HCC. The largest and previously untreated lesion in each patient was analyzed. Contrast-enhanced ultrasound (US) was performed to evaluate the presence of arterial blood flow in the lesion before baseline MRI. A diagnostic US system (SSA-790A, Aplio XG; Toshiba Medical Systems Corporation, Otawara, Japan) with a 3.75-MHz convex transducer was used. A second-generation US contrast agent (Sonazoid; Daiichi-Sankyo, Tokyo, Japan) was injected as a 0.5-mL bolus into an antecubital vein followed by a 10-mL saline flush at 1 mL/s.

### MRI Protocol

MR imaging was performed with a 1.5-Tesla scanner 32-channel coil system (Avanto, Siemens Medical Systems, Erlangen, Germany) with a peak slew rate of 200 T/m/s. MRI sequences were subjected to T1-weighted imaging, T2-weighted imaging, and DWI.

T1-weighted images were acquired using the following sequence parameters: gradient echo sequence; repetition time: 125 ms; dual echo time; opposed phase: 2.38 ms; in phase: 4.76 ms; flip angle: 75°; matrix size: 320 × 126; field of view: 400 × 454 mm; slice thickness: 5 mm; receiver bandwidth: 470 Hz/pixel; acquisition time: 13 s.

T2-weighted images were acquired during breath-holding using the following sequence parameters: turbo spin-echo sequence; repetition time: 3,980 ms; echo time: 95 ms; flip angle: 150°; matrix size: 320 × 135; field of view: 400 × 454 mm; slice thickness: 5 mm; receiver bandwidth: 260 Hz/pixel; acquisition time: 20 s. DW images were acquired using the following sequence parameters: spin echo based echo-planar imaging; repetition time: 1,200 ms; echo time: 63 ms; flip angle: 90°; matrix size: 110 × 110; field of view: 400 × 454 mm; 1 averaging; slice thickness: 5 mm; receiver bandwidth: 921 Hz/pixel; fat suppression; spectral pre-saturation with inversion recovery; acquisition time: 20 s; *b*-values: 0, 50, 100, 150, 200, 400, and 800 s/mm^2^. DWI was performed during breath-holding.

Tumors were evaluated by MRI at baseline, and at 1, 2, and 4 weeks after sorafenib treatment.

### Follow-up

CT or MRI was performed before the start of sorafenib therapy, at 1 month and every 2 months thereafter. Dynamic CT was performed using either a 16-detector row or 64-detector row CT scanner. Iohexol 300 (Omnipaque 300, Daiichi-Sankyo) was injected over 30 s [12]. The amount of contrast agent used was 600 mgI/kg [13]. The arterial-dominant phase was obtained using a monitor scan; following this the portal-dominant phase and equilibrium phase were obtained. Dynamic MRI was performed using gadoterate meglumine (Magnescope, Guerbet) or gadolinium-ethoxybenzyl-diethylenetriamine pentaacetic acid (Primovist, Bayer). Magnescope (0.1 mmol/kg) was injected at 2 mL/s and Primovist (0.025 mmol/kg) was injected at 1 mL/s. Monitor scan was performed by first obtaining the arterial-dominant phase and then the portal-dominant and equilibrium phases. We evaluated the curative effects using dynamic CT or dynamic MRI at baseline, and after 1, 2, and 4 weeks of sorafenib treatment. We evaluated the curative effect by modified RECIST [3]. Curative effects were divided into 2 groups, namely, responders (complete response, partial response, and stable disease) and non-responders (progressive disease).

### Changes in signal strength

Changes in signal strength of the lesions on T1-weighted and T2-weighted imaging were evaluated by 2 radiologists who had 2 and 24 years of experience, by consensus reading. They compared the signal strengths and homogeneity of the lesions at baseline with those after 1, 2, and 4 weeks of sorafenib treatment, and they recorded whether a difference was present or absent.

### Calculation of IVIM parameters

The IVIM model is considered to provide the pure molecular diffusion (D) separately from the blood microcirculation (proportion of blood microcirculation [PF] and pseudo-diffusion coefficient [D*]), when multiple *b*-values are obtained, from low *b*-values (<200 s/mm^2^) to high *b*-values (>200 s/mm^2^) [14]. IVIM parameters were calculated using the following formula [14]:

1 $$ {\mathrm{S}}_{\mathrm{b}}/{\mathrm{S}}_0=\mathrm{f}\times \exp \hbox{-} \left\{\left(\mathrm{D}*+\mathrm{D}\right)\times \mathrm{b}\right\}+\left(1 - \mathrm{f}\right)\times \left\{-\mathrm{D}\times \mathrm{b}\right\} $$

D: true diffusion coefficient (DC); D*: pseudo-diffusion coefficient; f: perfusion fraction (PF); S_b_, S_0_: signal intensity with and without the application of the diffusion gradient, respectively.

The 2-step fitting procedure was adopted to determine PF, D, and D*, required because of the high dispersion and limited sampling of DWI signals at low *b*-values (*b* < 200 s/mm^2^). Values of D were estimated from signal intensity data at high *b*-values (*b* > 200 s/mm^2^). Considering that D* is significantly greater than D, its effect on signal decay can be neglected for *b*-values greater than 200 s/mm^2^. Eq. (1) can be simplified and the D can be obtained using only *b*-values equal to or greater than 200 s/mm^2^, with the following simple linear fit equation:

2 $$ {\mathrm{S}}_{\mathrm{b}}/{\mathrm{S}}_0= \exp\ \left(-\mathrm{b}\mathrm{D}\right) $$

After determination of the D-value using Eq. (2), PF and D* can be processed using a nonlinear least squares estimate based on Eq. (1). The apparent diffusion coefficient (ADC) was obtained using all *b*-values by simple linear fitting as in Eq. (2). IVIM parameters were calculated using freely available software at the website (http://yamarad.umin.ne.jp/ivim/simplex.html). The IVIM data were constrained as follows: 0 < PF < 1, 0 < D < D* < 1 mm^2^/s, 0 < ADC.

### Statistical analysis

IVIM parameters were expressed as means ± standard deviations. Changes in the signal strengths of T1-weighted and T2-weighted images of the responder group and the non-responder group were statistically analyzed using the chi-squared test. The differences in IVIM parameters between the responder group and the non-responder group were statistically analyzed using the Mann–Whitney *U* test and Fisher exact test. In addition, differences in IVIM parameters between pre-treatment and post-treatment were evaluated using the Friedman test. A *p*-value less than 0.05 was considered to indicate a statistically significant difference between 2 groups. When a significant difference was observed, the cut-off value was determined by receiver operating characteristic analysis, and then sensitivity and specificity were calculated. All statistical analyses were performed using SPSS statistics software (version 22, SPSS) for Microsoft windows.

## Results

Six patients (67 %) had at least 1 non-permissible value of PF or D* within the 4 consecutive examinations. Six patients were classified as responders (Complete Response: 1; Stable Disease: 5), and 3 patients were classified as non-responders. Detailed information of the patients is described in Table 1. The sizes of the lesions did not significantly change in both the responder and non-responder group, although the lesions in the non-responder group tended to increase in size. There were no remarkable signal changes between before and after treatment on T1-weighted and T2-weighted imaging in both the responders and the non-responders.

**Table 1:** Patient characteristics

	Responder	Non-responder
Age (yrs; mean ± SD)	72.2 ± 2.7	61.3 ± 13.1
Sex		
Men	5	2
Women	1	1
Cause of disease		
Hepatitis B	0	1
Hepatitis C	6	2
BCLC stage		
B	4	1
C	2	2
Previous therapy		
Surgery	1	0
TAE/TACE/RFA	4	2
None	1	1
Tumor size (mm; mean ± SD)		
Baseline	30.7 ± 22.1	33.5 ± 14.1
1 week	30.3 ± 21.8	35.0 ± 14.6
2 weeks	31.0 ± 20.7	37.3 ± 16.7
4 weeks	31.5 ± 19.4	38.5 ± 15.9
Treatment		
Sorafenib (initial dose)	200 mg twice daily	200 mg twice daily

*BCLC stage* Barcelona Clinic Liver Cancer stage, *TAE* transcatheter arterial embolization, *TACE* transcatheter arterial chemoembolization, *RFA* radiofrequency ablation therapy

**Fig. 1:**
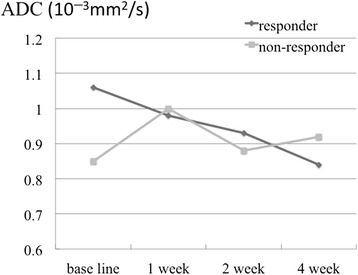
Apparent diffusion coefficients (ADCs) of the responders and non-responders at baseline, and after 1, 2, and 4 weeks of sorafenib treatment. At baseline, ADC values in the responder group were higher than those in the non-responder group; however, the difference was not statistically significant

**Fig. 2:**
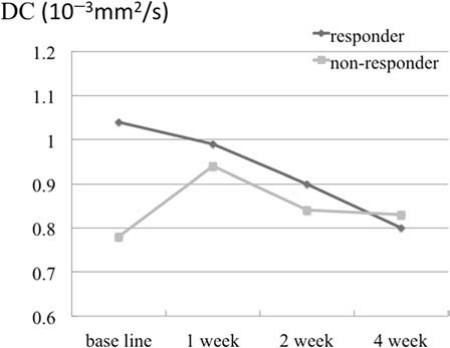
True diffusion coefficients (DCs) of the responders and non-responders at baseline, and after 1, 2, and 4 weeks of sorafenib treatment. DC values of the responder group were significantly higher than those of the non-responder group at baseline

**Fig. 3:**
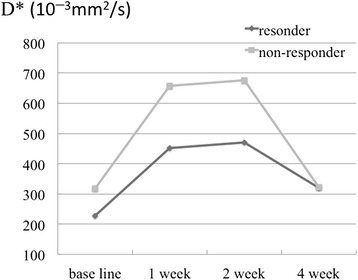
Pseudo-diffusion coefficients of the responders and non-responders at baseline, and after 1, 2, and 4 weeks of treatment. No significant changes or differences were observed between the 2 groups

**Fig. 4:**
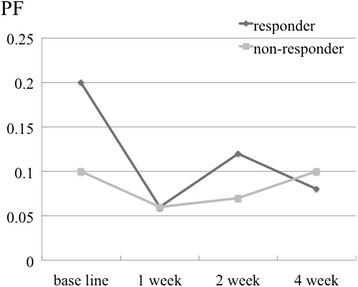
Perfusion fractions of the responders and non-responders at baseline, and after 1, 2, and 4 weeks of sorafenib treatment. No significant changes or differences were observed between the 2 groups

The IVIM parameters of ADC, D, D*, and PF in the responders and non-responders at baseline, and after 1, 2, and 4 weeks of treatment are shown in Table 2 and Figs. 1, 2, 3 and 4.

**Table 2:** IVIM parameters of responders and non-responders

	ADC (10^−3^ mm^2^/s)		DC (10^−3^ mm^2^/s)		D* (10^−3^ mm^2^/s)		PF	
	Responder	Non-responder	Responder	Non-responder	Responder	Non-responder	Responder	Non-responder
Baseline	1.06 ± 0.21	0.85 ± 0.04	1.04 ± 0.23	0.78 ± 0.06	228 ± 392	317 ± 274	0.20 ± 0.28	0.10 ± 0.08
1 week	0.98 ± 0.25	1.00 ± 0.05	0.99 ± 0.38	0.94 ± 0.20	452 ± 376	657 ± 555	0.06 ± 0.04	0.06 ± 0.04
2 weeks	0.93 ± 0.16	0.88 ± 0.33	0.90 ± 0.25	0.84 ± 0.32	470 ± 441	676 ± 272	0.12 ± 0.11	0.07 ± 0.08
4 weeks	0.84 ± 0.11	0.92 ± 0.33	0.80 ± 0.08	0.83 ± 0.23	321 ± 389	323 ± 276	0.08 ± 0.06	0.10 ± 0.12

*ADC* apparent diffusion coefficient, *DC* true diffusion coefficient, D* pseudo-diffusion coefficient, *PF* perfusion fraction

The ADCs of the responders at baseline, and after 1, 2, and 4 weeks of treatment were 1.06 ± 0.21, 0.98 ± 0.25, 0.93 ± 0.16, and 0.84 ± 0.11 (10^−3^ mm^2^/s), respectively, and of the non-responders were 0.85 ± 0.04, 1.00 ± 0.05, 0.88 ± 0.33, and 0.92 ± 0.33 (10^−3^ mm^2^/s), respectively. A statistically significant difference was not detected between the responders and the non-responders. (*p* = 0.095, 0.548, 1.00, and 0.905 at baseline, and after 1, 2, and 4 weeks of treatment, respectively). The DC of the responders at baseline, and after 1, 2, and 4 weeks of treatment were 1.04 ± 0.23, 0.99 ± 0.38, 0.90 ± 0.25, and 0.80 ± 0.08 (10^−3^ mm^2^/s), respectively, and of the non-responders were 0.78 ± 0.06, 0.94 ± 0.20, 0.84 ± 0.32, and 0.83 ± 0.23 (10^−3^ mm^2^/s), respectively. The DC of responders at baseline was significantly higher than that of the non-responders (*p* = 0.048, 1.00, 0.905, and 0.714 at baseline, and after 1, 2, and 4 weeks of treatment, respectively). The sensitivity and specificity, when a DC of 0.8 (10^−3^ mm^2^/s) or higher was considered to be a responder, were 100 % (95 % CI: 83, 100) and 67 % (95 % CI: 27.2, 66.7), respectively. The D*s of responders at baseline, and after 1, 2, and 4 weeks of treatment were 228 ± 392, 452 ± 376, 470 ± 441, and 321 ± 389 (10^−3^ mm^2^/s), respectively, and of the non-responders were 317 ± 274, 657 ± 555, 676 ± 272, and 323 ± 276 (10^−3^ mm^2^/s), respectively. There were no significant differences between the 2 groups (*p* = 0.381, 0.714, 0.548, and 1.00 at baseline, and after 1, 2, and 4 weeks of treatment, respectively). The PFs of the responders at baseline, and after 1, 2, and 4 weeks of treatment were 0.20 ± 0.28, 0.06 ± 0.04, 0.12 ± 0.11, and 0.08 ± 0.06 (10^−3^ mm^2^/s), respectively, and of the non-responders were 0.10 ± 0.08, 0.06 ± 0.04, 0.07 ± 0.08, and 0.10 ± 0.12 (10^−3^ mm^2^/s), respectively. There were no significant differences between the 2 groups (*p* = 0.905, 1.00, 0.905, and 1.00 at baseline, and after 1, 2, and 4 weeks of treatment, respectively).

Regarding changes in each parameter with treatment, the responders showed a decrease in the ADC and DC, whereas the non-responders did not. However, these changes were not statistically significant (*p* = 0.102 and 0.719 for ADC, and *p* = 0.100 and 0.334 for DC in the responder and non-responder group, respectively). The responders showed a decrease in PF at 1 week after treatment compared with the baseline, but this was not statistically significant (*p* = 0.978 and 0.801 in the responder and non-responder group, respectively). Furthermore, we did not observe a statistically significant difference in D* (*p* = 0.261 and 0.801 in the responder and non-responder group, respectively).

## Discussion

In our study, DC values in the responder group were significantly higher than those in the non-responder group at baseline, suggesting that it is possible to predict therapeutic outcome before the initiation of treatment. In addition, at baseline, ADC values of the responder group were higher than those of the non-responder group, although this difference was not significant. Woo et al. reported that the histological grade of HCC correlated more strongly with the DC than the ADC [15]. Because the ADC includes not only pure diffusion, but also perfusion as compared with DC, its interpretation may be complicated. They reported that the DC of patients with high-grade HCC was significantly lower than that of patients with low-grade HCC. It was also reported that favorable treatment results were obtained with sorafenib in patients with histologically well-differentiated tumors. Furthermore, the degrees of differentiation of tumors were shown to correlate with their expression levels of vascular endothelial growth factor (VEGF), i.e., high expression levels of VEGF indicated well-differentiated HCC [16]. We believe that the results of our present study reflect the results of this previous study [17].

The DC and ADC values showed a decrease in the responders with treatment, but this was not significant, and is consistent with previous reports [6, 7]. Schraml et al. reported that these changes are caused by bleeding [5]; however, we did not detect any bleeding, consistent with the report by Lewin et al. [7]. They reported that the ADC was increased at 2–3 months after treatment because of necrotic changes. We did not find any obvious changes on the images, such as those reflecting necrosis, because we only evaluated the patients up to 4 weeks of treatment.

We observed a decrease in the PF after 1 week of treatment in the responders. On the other hand, Lewin et al. reported an increase in the PF after 2 weeks of treatment [7]. Because sorafenib inhibits tumor angiogenesis, it causes the disruption and normalization of tumor vessels [18]. This normalization of tumor blood vessels suppresses permeability, resulting in a decrease in the pressure of the tumor tissue. Lewin et al. described the cause of the increased PF as an increase in the perfusion rate by normalization of tumor blood vessels. In our present study, the factor that differed from the study of Lewin et al. was the scanning periods. In addition, the method of calculation of the PFs was also different. It has been reported that PFs and D*s have poor reproducibility [19]. Therefore, it may be useful to scan many low *b*-values to obtain a stable PF value. We measured a total of 7 *b*-values (0, 50, 100, 150, 200, 400, and 800 s/mm^2^), whereas Lewin et al. measured a total of 4 (0, 200, 400, and 800 s/mm^2^). In this regard, our results may be more reliable.

Our study has several limitations. The first limitation is the small number of patients studied. High sensitivity and specificity of differentiation between responders and non-responders was found in our study. However, these results might be an overestimation because of the small number of subjects. The study should be repeated with a larger number of patients in the future. The second limitation is that some cases showed poor fitting of IVIM. When such cases occurred, outliers were removed from the measurements. In other studies, scanning by the appropriate *b*-values and other techniques, such as Bayesian fitting, were used to obtain better fitting [20, 21]. In the future, other methods to improve the fitting should be tested. The third limitation is the lack of evaluation of reproducibility. A previous report stated that D* and PF showed poor reproducibility, whereas DC showed relatively high reproducibility [19]. Therefore, we believe that the conclusion of our study is reliable. The fourth limitation is that we used breath-holding DWI. This technique is faster than respiratory-triggered DWI but has the problem of a lower signal-to-noise ratio. However, Kim et al. reported that ADCs calculated from breath-holding DWI were more reproducible than those from respiratory-triggered DWI [22]. Furthermore, respiratory-triggered DWI requires longer acquisition times, and is prone to misregistration, potentially leading to an inaccurate ADC map [23]. Therefore, we believe that the breath-holding technique was adequate for performing routine examinations.

## Conclusions

In conclusion, our results suggest that the DC obtained by IVIM MRI may be useful as a biomarker for predicting the therapeutic effects of sorafenib for HCC.
